# Supplementary material for: A Review of Clinical Outcomes, Owner Understanding and Satisfaction following Medial Canthoplasty in Brachycephalic Dogs in a UK Referral Setting (2016–2021)
Source: Animals (Basel). 2023 Jun 19;13(12):2032. doi: 10.3390/ani13122032 (PMC10295752; doi:10.3390/ani13122032)
Supplement: Supplementary file 1 [file animals-13-02032-s001.zip › animals-2406247-supplementary.pdf]

Supplementary

Table S1. Mucoid discharge and conjunctival hyperaemia of dogs with brachycephaly recommended medial canthoplasty from 2016-2021 under referral veterinary care at the Queen Mother Hospital for Animals, Royal Veterinary College, UK.

	**Total study population (pre recommended MC*)**				**Atopic dermatitis**				**Non atopic dermatitis**			
	**Ulcerated**		**Non-ulcerated**		**Ulcerated**		**Non-ulcerated**		**Ulcerated**		**Non-ulcerated**	
	**n/X**	**%**	**n/X**	**%**	**n/X**	**%**	**n/X**	**%**	**n/X**	**%**	**n/X**	**%**
Mucoid ocular discharge	12/25	48.0	29/246	11.8	1/4	25.0	12/38	31.6	2/21	9.5	60/208	28.8
Conjunctival hyperaemia	8/25	32.0	21/246	8.5	1/4	25.0	15/38	39.5	3/21	14.3	46/208	22.1

MC* Medial canthoplasty
